# Basic Characteristics of a Macroscopic Measure for Detecting Abnormal Changes in a Multiagent System

**DOI:** 10.3390/s150409112

**Published:** 2015-04-17

**Authors:** Tetsuo Kinoshita

**Affiliations:** Research Institute of Electrical Communication, Tohoku University, Katahira 2-1-1, Aoba-ku, Sendai 980-8577, Japan; E-Mail: kino@riec.tohoku.ac.jp; Tel./Fax: +81-22-217-5415

**Keywords:** macroscopic behavioral model, variance of fluctuation, behavioral monitoring, multiagent system, empirical study

## Abstract

Multiagent application systems must deal with various changes in both the system and the system environment at runtime. Generally, such changes have undesirable negative effects on the system. To manage and control the system, it is important to observe and detect negative effects using an appropriate observation function of the system’s behavior. This paper focuses on the design of this function and proposes a new macroscopic measure with which to observe behavioral characteristics of a runtime multiagent system. The proposed measure is designed as the variance of fluctuation of a macroscopic activity factor of the whole system, based on theoretical analysis of the macroscopic behavioral model of a multiagent system. Experiments are conducted to investigate basic characteristics of the proposed measure, using a test bed system. The results of experiments show that the proposed measure reacts quickly and increases drastically in response to abnormal changes in the system. Hence, the proposed measure is considered a measure that can be used to detect undesirable changes in a multiagent system.

## 1. Introduction

A multiagent system (MAS) consists of agents who operate cooperatively as a team and handle problem-solving tasks according to their unique features relating to autonomy, reactivity, and social ability. At the runtime of an MAS, the system’s behavior fluctuates according to various changes in both the system and system environment. Because these changes generally have undesirable negative effects on the system, the system has to deal with the changes to maintain its behavior and performance. It is desirable to detect negative effects quickly using appropriate monitoring functions of the system’s behavior before fatal changes to the system occur. To develop monitoring functions for various information systems, measures for observing the behavior of systems have been investigated. Moreover, quantitative and qualitative measures such as the quality of service (QoS) have been developed for various information systems aiming to realize user-oriented services. However, it appears difficult to use these measures to detect abnormal situations for a system because observed measures such as the QoS fluctuate immediately after various changes within the system itself. Useful methods for detecting abnormal situations according to the system’s changes before the QoS degrades have yet to been established. Hence, the development of a new measure and/or measurement method that predicts the occurrence of abnormal changes is an important problem to solve to avoid unrecoverable situations of a system.

Focusing on the problem above, this paper proposes a new macroscopic measure defined using the macroscopic activity factor of an MAS according to theoretical analysis of the behavioral model of the MAS. The proposed measure is defined as the variance in fluctuation of the activity factor of the MAS. The analysis of the behavioral model reveals that the proposed measure increases drastically when the activity factor degrades. Therefore, this paper assumes a working hypothesis that clues that can reveal undesirable changes in the system are unusual increases in the proposed measure. Basic characteristics of the proposed measure are then investigated in experiments using a test bed system of a hierarchical MAS. The experiments show that the proposed measure reacts quickly in response to a system’s abnormal changes in terms of a drastic rise in the observed values as suggested by the working hypothesis above.

The remainder of the paper is organized as follows: Section 2 explains the background and motivation of the study. Section 3 defines a behavioral model of the MAS from a macroscopic viewpoint and proposes a new macroscopic measure using this model. The proposed measure is defined as the variance of fluctuation of the macroscopic activity factor of the MAS. Section 4 designs a measurement function for a test bed system using the proposed measure, and realizes a test bed system using a repository-based multiagent framework. The results of experiments conducted using the test bed system are presented to demonstrate basic characteristics of the proposed measure. Finally, Section 5 concludes the paper.

## 2. Background and Motivation

It is difficult to presume the nondeterministic behavior of an MAS at runtime because the properties and functions of agents are affected directly or indirectly by various changes in the system’s runtime conditions and environment. These changes occasionally have undesirable effects and can even cause catastrophic damage to the runtime system. It is thus necessary to develop effective methods and functions to maintain the system’s behavioral characteristics in response to such unusual situations.

Studies, such as those on resilient systems, have required a system to be proactive and able to anticipate, monitor and respond to environmental change or unexpected emerging threats. To realize such capabilities, Balchanos *et al.* [1] proposed a set of problem-dependent metrics that capture a resilient system’s ability to perform essential functions of a naval cooling network. The risks faced by MASs, which can cause serious breakdowns in systems’ operations, have been studied in contexts such as the trust management of MASs [2]. Self-protecting software systems have been studied as a class of autonomic systems capable of detecting and mitigating security threats at runtime [3]. Kaindl *et al.* [4] proposed a description model called self-representation for agents that represents both the software and hardware of mechatronic components in flexible automation systems. Moreover, Rahimian *et al.* [5] proposed a method of preserving the structural controllability of multiagent networks in the event of simultaneous failures using different quantitative measures of reliability. The above studies provided concepts and methods related to the design of new measures, which is the aim of the present paper.

Qualitative/quantitative measures or indexes have been studied in many technical domains, and schemes on the QoS [6] are adopted in various information systems including MASs. For instance, a multiagent application system, which operates adaptively in response to changes in both the user’s requests and the system environment, has been realized using problem-oriented QoS parameters of multimedia communication services [7,8]. Furthermore, Gutierrez *et al.* [9,10] studied an interesting method of detecting undesirable patterns of communication in MASs based on metrics for the observation of the performance of agents. The proposed metrics are defined as microscopic measures, which are calculated using various parameters representing agents’ activities. These metrics are different from the QoS but relate strongly with the QoS of an MAS, and they are used to detect undesirable behavioral patterns of an MAS and thus assist designers of an MAS. The results of measurement are used to find out undesirable pattern of communication, which should be improved using the detailed design information of the system. On the other hand, our measure is defined as a macroscopic index based on the macroscopic activity factor of the whole system. The purpose of the proposed measure is to make the system’s users aware of changes of the behavioral situations of the whole system as soon as possible. Moreover, the macroscopic activity factor can be observed by a relatively simple method, which does not utilize the microscopic metrics like QoS parameters of the system’s components.

Furthermore, it should be noted that undesirable changes in QoS parameters are observed only after the system has been altered. It is difficult to sense occurrences of undesirable changes in the system before the system falls into abnormal situations using QoS schemes only. Therefore, in our previous work [11], an index named the margin of potential capability (MoC) was designed to observe the degree of maneuverability of QoS parameters of an agent-based application system. Using the predefined threshold values of the MoC, degradations of maneuverability of the system can be detected and recovery processes relating to a system’s behavioral properties can be activated before the system goes down. However, the MoC is calculated using the observed QoS parameters, and the system thus cannot predict the occurrence of changes in the system using the MoC. If we want to avoid fatal situations like a system going down, we have to devise a new measure that is similar to leading indicators in the field of economics to predict undesirable changes of the system.

We thus introduce a macroscopic behavioral model of the MAS in this paper, and design a new observable measure based on theoretical analysis using this model. There have been studies on the design of metrics that are used in model-based methods to detect and analyze abnormal parts and/or faults in MASs. For instance, Franch [12] investigated a framework for the analysis of predictability of agent-oriented models written in the i* language using indicators to define metrics that measure model properties. Moreover, Mani *et al.* [13] proposed a testing method that extracts potential deadlock information from the design models of an MAS. This method allows the testing of a multiagent manufacturing system for deadlocks while the system is under development. The models of above papers are defined to describe the functional specification of an MAS and used in the design stage of an MAS. These models are useful in the design and implementation of MASs. In contrast, our behavioral model provides theoretical foundation of the design of a macroscopic measure to detect abnormal changes of an MAS, but does not aim to support the development of various MASs.

Against the background discussed above, this paper proposes a new macroscopic measure based on both the analysis of the theoretical behavioral model and experiments conducted using the test bed system of the MAS.

## 3. Method of Detecting Changes in a Behavioral Property of an MAS

### 3.1. Definition of the Macroscopic State Transition Equation of an MAS

**Figure 1:**
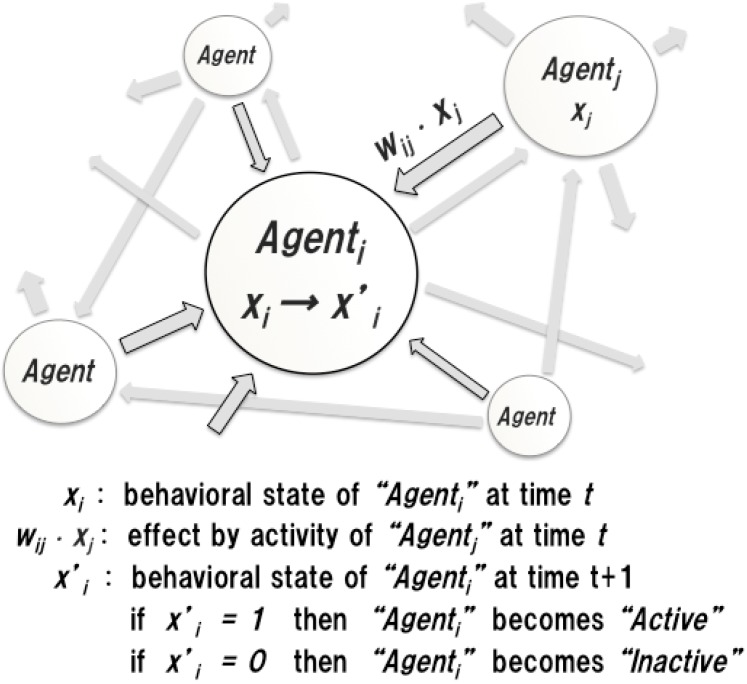
Model of a multiagent system.

To express the macroscopic behavior of an MAS, the structure and functions of the system are modeled using a macroscopic state transition equation. Let the number of agents in a system be *n*. An agent *A_i_* in the system has its own behavioral state *x_i_*, which represents the degree of activity of *A_i_* at time *t*. When *A_i_* works cooperatively with another agent *A_j_* with a behavioral state *x_j_*, behavioral states of *A_i_* are affected by actions of *A_j_* directly and/or indirectly, and vice versa. When there are many tasks in the system, many agents become active to process tasks. For instance, if *A_j_* works actively and its behavioral state is high, the possibility of increasing the degree of activity of *A_i_* increases. Hence, in this model, a behavioral state of *A_j_* at time *t* is determined according to various effects of other agents. The degree of positive effects of *A_j_*, with respect to *A_i_* at time *t*, is specified by a parameter *w_ij_*, called the cooperation coefficient, as shown in Figure 1.

To simplify the behavioral model, let a behavioral state *x* be a binary variable. The behavioral state $x_{i}^{\prime }$ of *A_i_* at time *t* + 1 is defined using a step function:

(1) $$x_{i}^{\prime }=\;stp(u_{i})\;u_{i}=\sum_{j=1}^{n}w_{ij}\cdot x_{j}-h_{i},\;0\leq w_{ij}\leq 1\;x_{i}^{\prime }=\{\begin{array}{c} 1,\;u_{i}>0\\ 0,\;u_{i}\leq 0 \end{array}$$

Here, $h_{i}$ is called the threshold coefficient. This parameter specifies the degree of degradation of activity of an agent, due to the runtime situation. For instance, conflicts relating to resources of agents on a single platform and overheads of processing and communication of agents on distributed platforms can be considered causes of degradation. If the threshold coefficient of an agent is small, then the agent has enough room to work more actively.

Next, the macroscopic behavior of the MAS is defined using behavioral states of the system. Because an agent is modeled as a threshold element, the MAS can be viewed as a network of threshold elements like a neural network. Hence, the macroscopic behavioral model of the MAS is defined employing a similar method of artificial neural network modeling [14].

A macroscopic state of the MAS at time *t* is given as a set of behavioral states of agents and represented as $X=[x_{1},\;x_{2},\ldots ,\;x_{n}]$. Let *Z* be the degree of activity at time *t*. The degree of activity Z’ at time *t* + 1 is given by the function:

(2) $$Act\left(X\right)=\;\frac{1}{n}・\overset{n}{\underset{i=1}{\sum }}x_{i}^{\prime }$$

Let the variables *u*, *w*, and *h* be Gaussian variables. The ensemble average of $u_{i}$ (*i.e.*, $<u_{i}>$) and its variance ${\text{σ}}_{u}^{2}$ are given as:

$$<u_{i}>=\sum_{j}<w_{ij}>\cdot x_{j}\;-\;<h_{i}>$$

Hence:

(3) $$\bar{u}=\;n∙\bar{w}∙Z-\bar{h}$$

where $\bar{u}=<u_{i}>,\;\bar{w}=<w_{ij}>,\;\bar{h}=<h_{i}>$ and ${\text{σ}}_{u}^{2}=n\cdot {\text{σ}}_{w}^{2}+{\text{σ}}_{h}^{2}$.

On the other hand, the probability density function of a Gaussian variable *u* is given by:

$$\frac{1}{\sqrt{2\text{π}\;}∙{\text{σ}}_{u}}\cdot \exp\left\{-\frac{{\left(u-\bar{u}\right)}^{2}}{2{\text{σ}}_{u}^{2}}\;\right\}$$

Hence, the probability of $x_{i}^{\prime }$ = 1 is calculated as:

(4) p=Prob{ui>0}=∫0∞12π ∙σu∙exp{−(u−u¯)22σu2 }du  =∫−u¯σ∞12π⋅exp{−v22} dv

The ensemble average of $x_{i}^{\prime },$ $<x_{i}^{\prime }>,$ is given by:

$$<\;x_{i}^{\prime }>\;=p\cdot 1+\left(1-p\right)\cdot 0=\;p$$

Using the error function, the ensemble average $<x_{i}^{\prime }>$ is expressed as:

(5) $$\text{Φ}\left(u\right)=\int_{-\infty }^{u}\frac{1}{\sqrt{2\text{π}}}\cdot \exp\left\{-\frac{v^{2}}{2}\right\}\;dv$$

(6) <xi′> = Φ(u¯σu)

The variance of the degree of activity, ${\text{σ}}_{u}^{2}$, is given as:

$$Z^{\prime }=\;<x_{i}^{\prime }>$$

(7) $${\text{σ}}_{u}^{2}=\;<{\left|\;Act\left(X\right)\;-\;<x_{i}^{\prime }>\right|}^{2}>\;=\;\frac{1}{n^{2}}\cdot \underset{i}{\sum }Var(x_{i}^{\prime })$$

When *n* is large, Act(X) can be approximated by $<x_{i}^{\prime }>$, because $Var(x_{i}^{\prime })$ can be considered zero. Hence, $Z^{\prime }$ is expressed as:

(8) $$Z^{\prime }=\;\text{Φ}\left(Z\right)=\;\text{Φ}\left(C\cdot Z-\text{Θ}\right)$$

**Figure 2:**
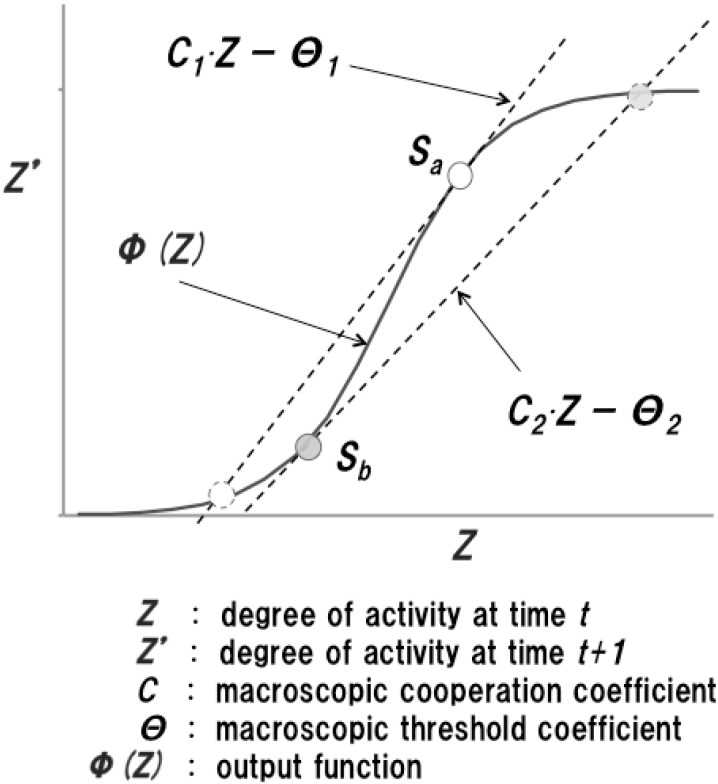
Image of unstable state of activity factor.

Here, C=n⋅w¯σu is the macroscopic cooperation coefficient and Θ=h¯σu is the macroscopic threshold coefficient. Formula (8) is the macroscopic state transition equation of the MAS, and the system’s characteristics can be manipulated by the system’s parameters *C* and Θ. Moreover, the macroscopic state transition function has hysteresis characteristics due to an error function. Hence, in the runtime system, it is expected that unstable states of the activity factor will emerge according to changes in the system’s parameters. An image of unstable states *S_a_* and *S_b_* with respect to different sets of parameters can be depicted as in Figure 2. Because an unstable state will jump to another state discontinuously, the behavioral characteristics of the system will also change suddenly at the unstable state.

### 3.2. Analysis of the Macroscopic Behavior of an MAS

To define a new measure that can be used to observe the behavior of the MAS as a whole, the macroscopic behavioral model of the MAS is constructed and analyzed using a master equation [15,16].

Let *N* be the number of agents of an MAS, $x_{i}$ be the state of an agent and $h_{i}$ be the threshold coefficient. The agent is in an active state if $x_{i}≧h_{i}$ and in an inactive state if $x_{i}<h_{i}$. Let *N_1_* be the number of active agents and *N_2_* be the number of inactive agents. The activity factor z at time *t* is then defined as:

(9) z= (N1−N2)N and N=N1+N2

An agent becomes active or inactive along with a process of the system’s behavior. Such a process is modeled as a stochastic birth–death process and represented by the following master equation. The following transitions of behavioral states of agents can occur for an initial state $\left(N_{1},\;\;N_{2}\right)$ at time *t*:

(10) $$(N_{1},\;N_{2})\to (N_{1}+1,\;N_{2}-1):\text{ an agent becomes active}$$

(11) $$(N_{1},\;N_{2})\to (N_{1}-1,\;N_{2}+1):\text{ an agent becomes inactive}$$

Let $W_{+,-}$ and $W_{-,+}$ be the ratios of transitions defined as:

(12) $$W_{+,-}\left(N_{1},\;N_{2}\;\to \;N_{1}+1,\;N_{2}-1\right)=N_{2}\cdot {\text{π}}_{1}(z)$$

(13) $$W_{-,+}\left(N_{1},\;N_{2}\;\to \;N_{1}-1,\;N_{2}+1\right)=N_{1}\cdot {\text{π}}_{2}(z)$$

Here, ${\text{π}}_{1}(z)$ is the transition rate for an inactive agent becoming active, and $\pi_{2}(z)$ is the transition rate for an active agent becoming inactive.

Let $P\;(N_{1},\;\;N_{2},\;t)$ be the probability density of finding a state $\left(N_{1},\;\;N_{2}\right)$ at time *t*. Considering Equations (10)–(13), the change of $P\;(N_{1},\;\;N_{2},\;t)$ at time *t* is defined as:

(14) $$P\;(N_{1},\;\;N_{2},\;t+1)=\;[1-\;N_{2}∙\pi_{1}\left(z\right)-\;N_{1}∙{\text{π}}_{2}\left(z\right)\;]∙P(\;N_{1},\;\;N_{2},\;t)\;+\;(N_{2}+1)∙{\text{π}}_{1}\left(z\right)\cdot P(\;N_{1}-1,\;\;N_{2}+1,\;t)\;+\;(N_{1}+1)∙{\text{π}}_{2}\left(z\right)\cdot P(\;N_{1}+1,\;\;N_{2}-1,\;t)$$

**Figure 3:**
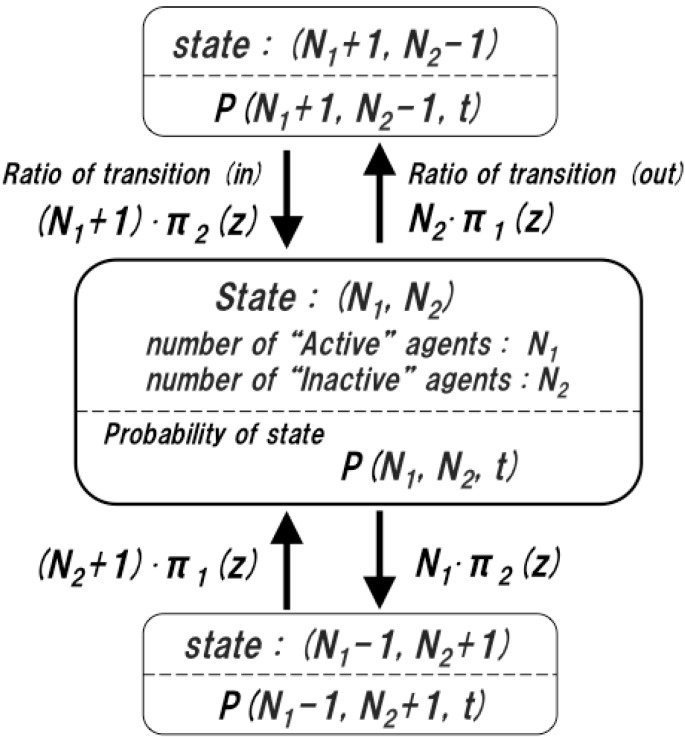
Transitions between state $\left(N_{1},\;\;N_{2}\right)$ and its adjacent states at time *t*.

As shown in Figure 3, first term of Equation (14) represents the effects that $\left(N_{1},\;\;N_{2}\right)$ changes to adjacent states $\left(N_{1}+1,\;\;N_{2}-1\right)$ and $\left(N_{1}-1,\;\;N_{2}+1\right)$, at time *t*. On the other hand, second and third terms represent the effects that $\left(N_{1}+1,\;\;N_{2}-1\right)$ and $\left(N_{1}-1,\;\;N_{2}+1\right)$ change to $\left(N_{1},\;\;N_{2}\right)$.

Equation (14) is rewritten as

(15) [P (N1,  N2, t+1)−P (N1,  N2, t)]t≅ ∂P(N1,  N2, t)∂t= −[ N2∙π1(z)+ N1∙π2(z) ]∙P( N1,  N2, t)     + (N2+1)∙π1(z)⋅P( N1−1,  N2+1, t)+ (N1+1)∙π2(z)⋅P( N1+1)

Using Equation (15), we define the master equation as:

(16) ∂P(N1,  N2, t)∂t=−[ N2∙π1(z)+ N1∙π2(z) ]∙P( N1,  N2, t)+ (N2+1)∙π1(z−ε)⋅P( N1−1,  N2+1, t)+ (N1+1)∙π2(z+ε)⋅P( N1+1,  N2−1, t)

Here, ε=2N and we assume ε ≪1.

Considering the relations:

N1=1ε⋅(1+z) and N2=1ε⋅(1−z)

which are derived from Equation (9), the master Equation (16) is rewritten as:

(17) ∂P(z, t)∂t=−[ 1ε⋅(1−z)∙π1(z)+1ε⋅(1+z)∙π2(z)]∙P(z, t)+ 1ε⋅(1−z+ε)∙π1(z−ε)⋅P(z−ε, t)+ 1ε⋅(1+z+ε)∙π2(z+ε)⋅P(z+ε, t)

Using a well-known procedure [17], the stochastic Equation (17) is equivalently transformed to the Fokker–Planck equation with respect to the fluctuation of activity factor, ξ, (see Appendix A):

(18) ∂P(ξ,t)∂t= − ∂∂z0∙[(1−z0)∙π1(z0)−(1+z0)∙π2(z0)]∙∂[ξ∙P(ξ,t)]∂ξ           + 12∙[(1−z0)∙π1(z0)+(1+z0)∙π2(z0)]∙∂2P(ξ,t)∂ξ2

Here, $z_{0}$ is the steady state of activity factor *z*, and the fluctuation ξ is defined as:

(19) $$\text{ξ}=\frac{1}{\sqrt{\text{ε}}}\cdot (z-z_{0})$$

Moreover, the evolution of *z*_0_ is given as (see Appendix A):

(20) ∂z0∂t= (1−z0)∙π1(z0)− (1+z0)∙π2(z0)

On the other hand, the evolution of the variance of the fluctuation of the activity factor is derived using Equation (18) (see Appendix B) as:

(21) ∂σξ2∂t=2∙∂[(1−z0)∙π1(z0)−(1+z0)∙π2(z0)]∂z0∙σξ2+ [(1−z0)∙π1(z0)+(1+z0)∙π2(z0)]

Using the results above, the characteristics of steady states of the activity factor are analyzed in the next section.

### 3.3. Macroscopic Measure Used to Observe the Behavioral Property of an MAS

In the steady state of the system, *z*_0_, the condition:

$$\frac{\partial z_{0}}{\partial t}=\frac{\partial \sigma_{\text{ξ}}^{2}}{\partial t}=0$$

is satisfied. Under this situation, the behavioral properties of an MAS are stable, and the system works smoothly. Using Equations (20) and (21), $z_{0}$ and $\sigma_{\text{ξ}}^{2}$ are calculated as:

(22) z0=[ π1(z0)− π2(z0) ][ π1(z0)+π2(z0) ]

(23) σξ2=−[(1−z0)∙π1(z0)+(1+z0)∙π2(z0)]2∙∂[(1−z0)∙π1(z0)−(1+z0)∙π2(z0)]∂z0

Moreover, we assume that ${\text{π}}_{2}\left(z_{0}\right)=1-{\text{π}}_{1}\left(z_{0}\right)$ and ${\text{π}}_{1}\left(z_{0}\right)=\text{π}\left(z_{0}\right)$, and $z_{0}$, $\text{π}\left(z_{0}\right)$ and $\sigma_{\text{ξ}}^{2}$ are calculated as:

(24) $$z_{0}=2∙\;\text{π}\left(z_{0}\right)-1$$

(25) π(z0)=(z0+1)2

(26) σξ2=−[(1−z0)∙π(z0)+(1+z0)∙π(z0)]2∙∂[(1−z0)∙π(z0)−(1+z0)∙π(z0)]∂z0=−[1+z0−2∙z0∙π(z0)]2・(2∙∂π(z0)∂z0−1)

When unusual changes occur in the runtime MAS, the system’s behavior becomes unstable temporary or permanently, and $\sigma_{\text{ξ}}^{2}$ fluctuates according to changes of activities of the MAS. It is obvious that $\sigma_{\text{ξ}}^{2}\to \;\infty$ when ∂π(z0)∂z0→12, that is, there exists a state where the variance of fluctuation is divergent.

**Figure 4:**
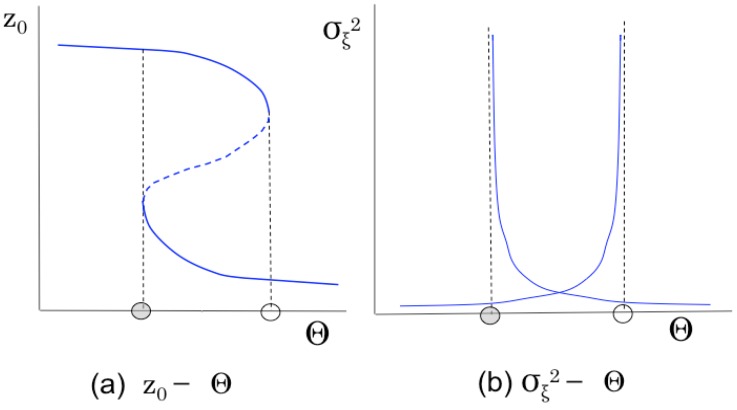
Image of transitions of z_0_ and $\sigma_{\text{ξ}}^{2}$ with respect to the parameter Θ.

As explained in Section 3.1, the macroscopic state transition function has hysteresis characteristics. Hence, an image of transitions of *z*_0_ and $\sigma_{\text{ξ}}^{2}$, with respect to the parameter Θ, can be depicted as in Figure 4. The variance of fluctuation gradually increases and finally diverges as the steady state approaches the point of discontinuous transition. Such a feature shows the possibility that the variance of fluctuation of the activity factor can be used as an observable measure of behavioral characteristics of the system. Furthermore, when an unusual increase in the variance of fluctuation of an activity factor is observed, it can be said that the system is in an unstable state and the system’s state is going to transit to another state in the near future, through abnormal changes of the system. Therefore, the variance of fluctuation of an activity factor of an MAS is proposed as a new measure for observing the behavior of a system, in this paper. The proposed measure is taken as a measurement function of an MAS. The specific measurement function of the test bed system is designed and implemented in the next section.

## 4. Experiments and Evaluation

### 4.1. Test Bed System and the Environment of Experiments

Experiments are conducted to test and validate the proposed measure using a test bed system. An important objective of the experiments is to explore basic properties and usefulness of the proposed measure using a real MAS in a real computer environment. The test bed system, which simulates typical behavior of multiagent applications such as a distributed monitoring system [18] and agent-based microgrid [19], developed in previous studies, has been realized as a simple hierarchical MAS using a repository-based multiagent framework [20,21].

**Figure 5:**
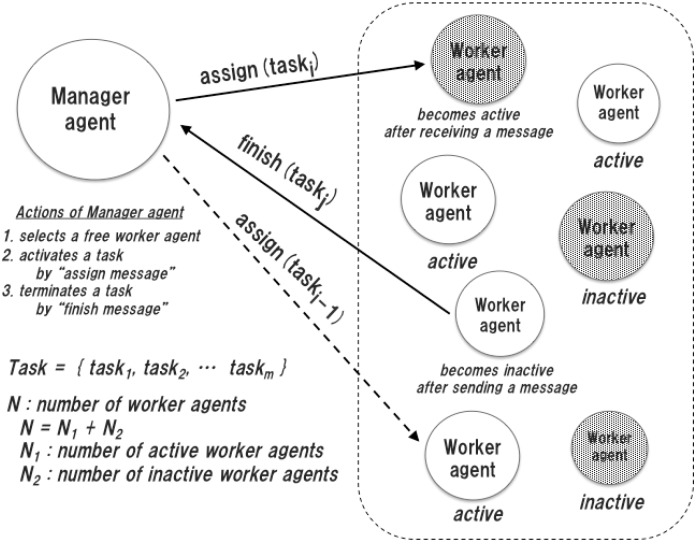
Structure of the test bed system.

The test bed system consists of one manager agent and many worker agents as shown in Figure 5. The manager agent generates a task, selects a worker agent, which is in the inactive state because it has no task to be processed, and assigns the task by sending an assignment message to the selected worker agent. In the test bed system, a task is designed as a pseudo task to make the selected worker agent active. If a task is assigned successfully, the manager agent considers that the selected worker agent is in the active state until receiving a finish message from the worker agent. From the viewpoint of the worker agent, the worker agent can accept and process one task at a time. The task process functions of worker agents are the same. The selected worker agent becomes active by accepting the assigned task, and stays active during the processing of the assigned task. When the assigned task is finished, the worker agent sends a finish message to the manager agent and becomes inactive again. Moreover, we introduce a random delay time before sending a finish message, to simulate the task process overhead of a worker agent. In this sense, the performance of each worker agent is different from each other.

The assigned task has information on the elapsed time, which specifies the required time to complete the task. An elapsed time of a task is set as a random value distributed around the specified average value. As mentioned above, we assign a random delay time of task process to each worker agent. Hence, the total processing time of the assigned task, called the duration time, is the sum of the elapsed time and delay time.

**Figure 6:**
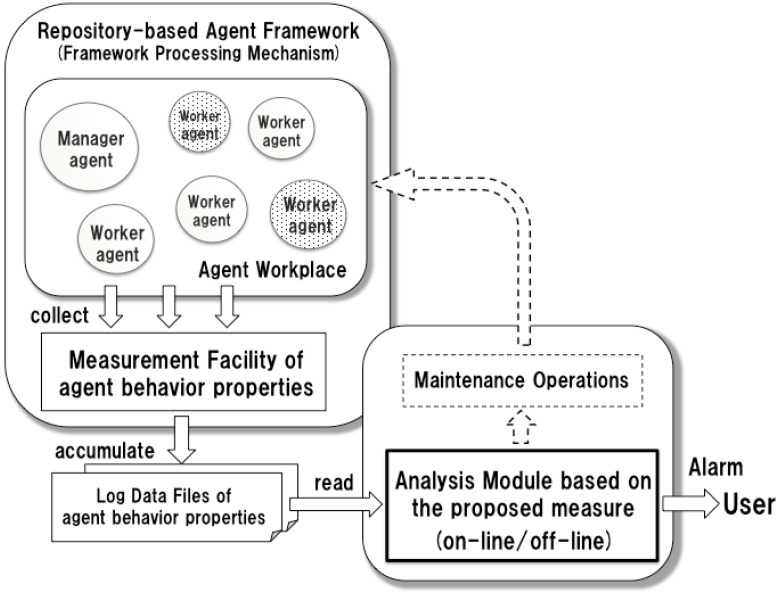
Configuration of the measurement environment.

All agents in the test bed system are designed and implemented as software agents using the repository-based agent framework and its interactive design environment (version: IDEA_1.3.1) [20,21]. The agent framework supports the development of multiagent applications, on both a single platform and distributed platforms. In the experiments, the test bed system is realized as a system running on a single computer (CPU: Core-i5 3.0 GHz, Memory: 4.0 GB, OS: Windows 7). For this architecture, each agent is directly and adversely affected by other agents’ workloads and minimizes the overhead of measurement throughout the system environment. The agent framework allows the measurement of the behavioral properties of agents. This facility collects data of runtime agents, such as the task processing time, the duration time, the messages processed by agents and the number of activated agents, and accumulates time series data as external log files, as shown in Figure 6. Moreover, the analysis module inspects behavioral situations of the system based on the proposed measure and the results are to be used by maintenance operations to be realized in future work. The function of the analysis module is discussed in Section 4.5.

### 4.2. Implementation of the Measurement Function

The proposed measure is defined according to the active agents in the test bed system. As shown in Figure 5, *N* is the total number of agents of the test bed system, and $n_{t}$ is the number of active worker agents at time T. According to the definition of the activity factor in Equation (9), the normalized activity factor $z_{t}\in \left[0,1\right]$ at time *T* is calculated in each unit time ∆T and is specified by the measurement facility of the agent framework:

(27) $$z_{t}=\frac{1}{2∙N}∙\left(2∙n_{t}-N+1\right)$$

The time series of the normalized activity factors, {$\;z_{1}$, $z_{2}$, ⋯, $z_{j}$, ⋯, $z_{t}$} is then given at time *T*. Here, $z_{j}$ is the *j*th element at time $T_{j}=T-(t-j)\times ∆T$. Next, the fluctuation of the activity factor is calculated as:

(28) $${\text{ξ}}_{t}=z_{t}-\frac{1}{L}∙\overset{L-1}{\underset{i=0}{\sum }}z_{t-i}$$

The variance of fluctuation of the activity factor, $v({\text{ξ}}_{t})$, is also calculated using part of a time series, {$\;{\text{ξ}}_{t-M+1}$, ⋯, ${\text{ξ}}_{t-1}$, $\;{\text{ξ}}_{t}$ }, as:

(29) $$v({\text{ξ}}_{t})=\frac{1}{M}∙\overset{M-1}{\underset{i=0}{\sum }}{\text{ξ}}_{t-i}^{2}-{\left(\frac{1}{M}∙\overset{M-1}{\underset{i=0}{\sum }}{\text{ξ}}_{t-i}\right)}^{2}$$

The smoothed data are useful for the detection of abnormal changes observed in the time series of $var({\text{ξ}}_{t})$. Hence, the moving average of the variance of fluctuation is calculated using part of a time series, {$v({\text{ξ}}_{t-M+1})$,  ⋯, $v({\text{ξ}}_{t-1})$, $v({\text{ξ}}_{t})$}, as:

(30) $$Var({\text{ξ}}_{t})=v({\text{ξ}}_{t})-\frac{1}{M}∙\overset{M-1}{\underset{i=0}{\sum }}v({\text{ξ}}_{t-i})$$

A new measurement function of the proposed measure is implemented using these equations as the design specification in the test bed system. In the implementation, the unit time ∆T is set to 2 s, and the parameters in above equations are set as *L* = 20 and *M* = 20.

### 4.3. Experiment 1 and Evaluation

#### 4.3.1. Setting of Experiment 1

The behavior of the test bed system in normal situations, in which the system does not change, is observed to study basic characteristics of the proposed measure. The structure and function of the normal test bed system do not change during the measurement in Experiment 1. The system consists of one manager agent and 150 worker agents. The elapsed time of a task is set as a random value selected between 18 and 22 s, and the average elapsed time is 20 s. The delay time of each task is also set as a random value, selected between 0 and 10 s, and the average delay time is 5 s. At the beginning of the experiment, to make worker agents active, the manager agent sends initialization messages to all worker agents, one by one, at intervals of 0.1 s, and the task assignment and processing begin. The log data of runtime agents are collected and accumulated in intervals of 0.01 s, until a time of 5760 s. Using the log data, the normalized activity factor and the variance of fluctuation are calculated in intervals of 2 s.

**Figure 7:**
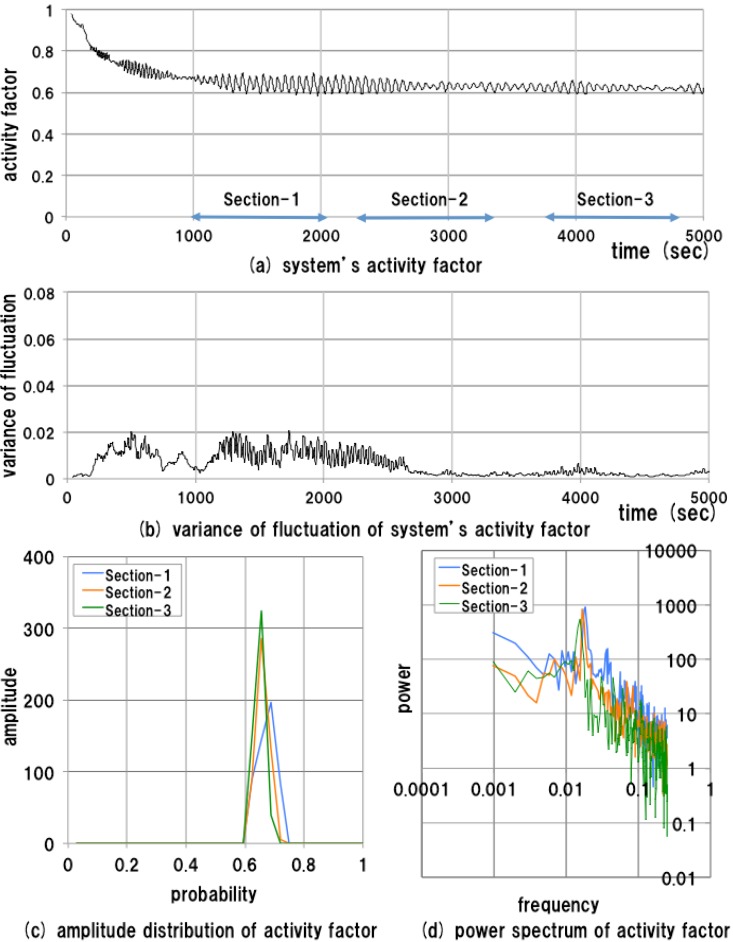
Results of measurements of the normal test bed system.

#### 4.3.2. Evaluation of Experiment 1

The results of measurement (*i.e.*, time series data of both the system’s activity factor and the variance of fluctuation of the activity factor) of a normal system are depicted in Figure 7a,b. In general, the amplitude distribution and power spectrum of the low-frequency region are useful for the observation of the features of time series data. Hence, three parts of the time series data specified as Section 1, Section 2 and Section 3 in Figure 7a, are selected and analyzed. Here, Section 1 is the steady-state region immediately after the transient region of initialization, while Section 2 and Section 3 are steady-state regions long after system initialization. We can specify these sections freely as non-overlapped sections in the steady-state region (600–5000 s). The length of each section is 1024 s. The periods of Section 1, Section 2 and Section 3 are set to 1000–2024, 2288–3312 and 3736–4760 s, respectively. The measurement results are shown in Figure 7c,d.

The temporal change in the proposed measure is no more than 0.02, indicating that the system behavior is stable; *i.e.*, the temporal transition of the system’s activity factor remains in a stable range of 0.6–0.7 after 1000 s. The amplitude distributions of activity factors in the three sections have almost the same average values, about 0.65, and the power spectra show almost the same features. The results of experiment 1 show that the behavioral property of the system is stable when the system does not change.

### 4.4. Experiment 2 and Evaluation

#### 4.4.1. Setting of Experiment 2

The purpose of Experiment 2 is to evaluate the proposed measure using a system that changes dynamically at runtime. This experiment simulates the unusual situations in which a system’s components halt gradually because of trouble in the system.

The setting of Experiment 2 is basically the same as that of experiment 1. However, the structure of the test bed system is changed at runtime by removing worker agents. Some worker agents are selected randomly and removed from the system at the time points given in Table 1. The system’s activity factor, in general, is worsened by the negative effects of structural and functional changes of the system. For the test bed system, the proposed measure can reveal the deterioration of macroscopic activity of the system but cannot classify causes of the deterioration. Therefore, structural changes of the system are adopted as a cause of deterioration.

**Table 1:** Setting of changes in the test bed system.

Time Point [s]	Number of Worker Agents to be Removed	Number of Agents of the System
600	1	150
1100	2	148
1600	4	144
2100	8	136
2600	16	120
3100	32	88

#### 4.4.2. Evaluation of Experiment 2

**Figure 8:**
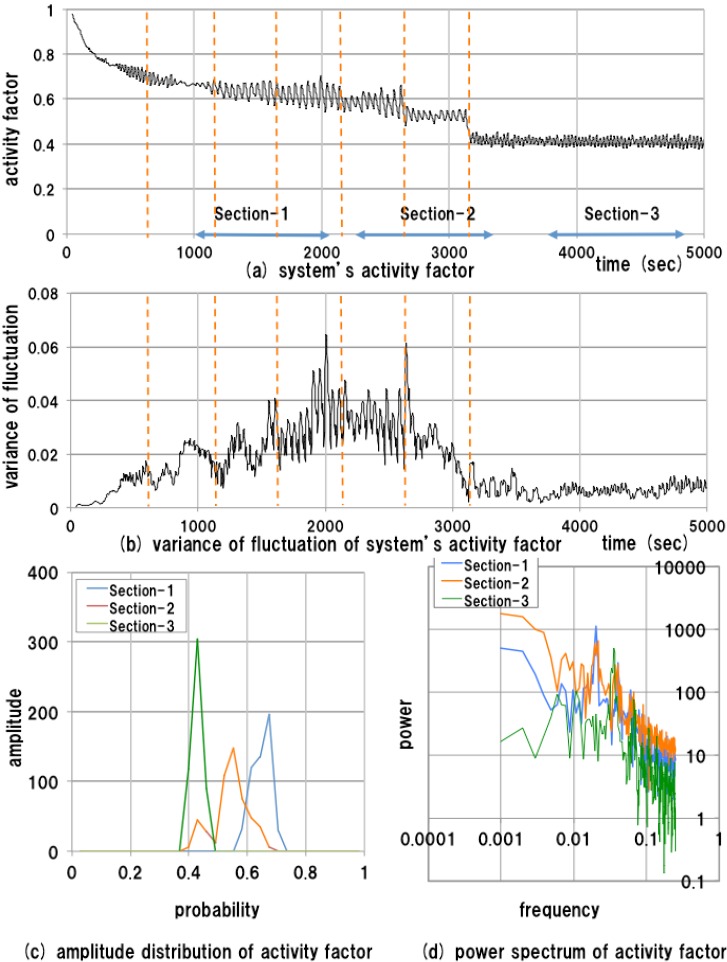
Results of measurements of the changed test bed system.

The temporal transitions of both the system’s activity factor and the variance of fluctuation of the system’s activity factor are shown in Figure 8a,b. The time points at which the worker agents are removed are indicated by dashed lines in Figure 8a,b. The amplitude distributions and the power spectra are calculated with respect to the sections specified for Experiment 1, and the results are shown in Figure 8c,d. However, in Experiment 2, Section 1, Section 2 and Section 3 correspond to regions of small changes, large changes, and no changes, respectively.

Interestingly, the temporal feature of the activity factor is similar to that in Experiment 1 until Section 2. The averages of activity factors in Section 1 and Section 2 fluctuate around 0.6 and the activity factor degrades a little according to changes in the system. The disturbance of the activity factor remains small until a discontinuous change at 3100 s. It thus appears difficult to detect abnormal situations for the system by observing only the transition of the activity factor, even when there are actual structural changes.

However, using the proposed measure, it becomes possible to detect abnormal situations according to an unusual increase in the proposed measure, in the early stages of abnormal situations of the system. The temporal feature of the variance of fluctuation of the activity factor is clearly different from that in Experiment 1. The variance of fluctuation becomes large gradually, according to changes in the system. At around the time point where the worker agents are removed, the proposed measure varies violently, and increases. The amplitude of the proposed measure becomes two or three times that for the unchanged system. As expected from theoretical analysis of the behavioral model in Section 3, an unusual increase in the proposed measure is observed in response to abnormal changes in the system.

Meanwhile, the variance of fluctuation becomes small after 2700 s. At this point, about 21% of the worker agents have been removed from the system, and it seems that the system’s behavioral characteristics transited to unusual states because of undesirable changes of the system. Obviously, the peak shifts can be observed in the amplitude distributions of Section 2 as shown in Figure 8c. Specifically, the single-peak distribution in Section 1 shifts to a double-peak distribution in Section 2, and then returns to a single-peak distribution in Section 3. Moreover, the features of the power spectrum of Section 2 resemble those of Section 1 whereas the power spectrum of Section 3 has different features compared with the other spectra.

The above result suggests that the behavioral characteristics of the initial system in Section 1 transformed to other characteristics via unstable states in Section 2. To inspect unstable behavioral situations in Section 2, the activity factor of the manager agent, who is responsible for controlling the whole system, is observed at the time corresponding to measurements in Experiments 1 and 2. The activity factor of the manager agent is a macroscopic measure defined as the rate of inference processing time per unit time. In the experiment, the unit time is set to 2 s.

**Figure 9:**
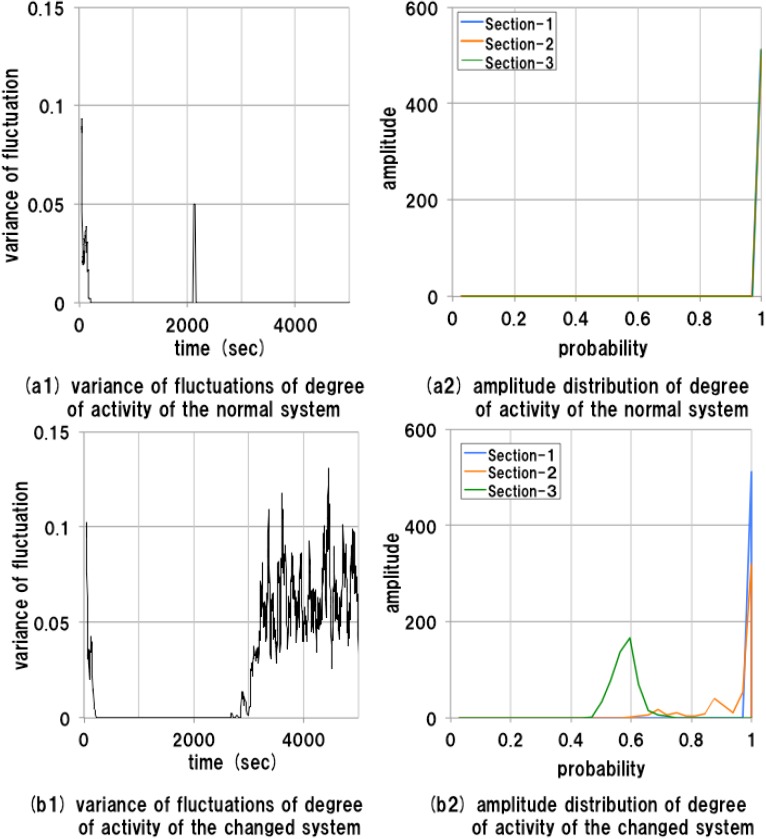
Results of measurements of the manager agent.

The result of measurement of the manager agent is shown in Figure 9. In the normal system, the manager agent works at almost 100% to control the whole system. The behavior of the manager agent is stable and the variance of fluctuation of the activity factor is always very small, as shown in Figure 9a1,a2. By contrast, in the changed system, the behavior of the manager agent is stable until the large change in the system at around 2600 s. However, immediately after this point, the behavior of the manager agent changes suddenly. The variance of fluctuation of the activity factor increases rapidly, and fluctuates violently, as shown in Figure 9b1. Moreover, as shown in Figure 9b2, there is a peak shift of the amplitude distribution in Section 2, and the activity of the manager agent reduces to about 60% of that of the normal system.

The negative effects of eliminating worker agents are observed immediately after the first change in Figure 8b. As the worker agents are removed step by step, the rate of responses of the worker agents decreases and it becomes difficult for the manager agent to find suitable worker agents to accept the assignment of new tasks. The idle time of the manager agent varies rapidly, depending on the fluctuating situation of the worker agents, and the average idle time also increases. The behavior of the manager agent becomes unstable states around 2600 s, as shown in Figure 9b1. The unstable states of both the manager agent and the whole system continue until the end of measurement, and the capability of the whole system also reduces. This result shows that the original behavioral characteristics of the test bed system have shifted to other characteristics at around 2600 s.

In Experiment 2, no point of divergence of the variance of fluctuation is observed because such a point corresponds to an unstable state in which the system cannot behave stably. However, it can be said that the basic feature of the proposed measure is confirmed by the results of experiments in this paper. When there are abnormal changes in the system, unusual changes of the fluctuation of the system’s activity factor emerge and the variance of fluctuation increases rapidly. Hence, the proposed measure (*i.e.*, the variance of fluctuation of the activity factor) can be considered a new observable measure with which to monitor the behavior of an MAS.

### 4.5. Considerations of the Observations of the Runtime System 

The results of the above experiments reveal that the proposed measure responds to undesirable changes in an MAS. Hence, a mechanism that provides an alarm in the event of changes in a runtime system can be realized using the proposed measure. In this section, an idea for the design of such an alarm mechanism is discussed.

As shown in Figure 6, the alarm mechanism will be realized as part of the function of the analysis module, which can work in on-line or off-line mode. In the off-line mode adopted in the experiments of this paper, various analyses of time series data can be conducted using all data. However, the alarm cannot be raised at the runtime of the system. In the test bed system, the measurement facility of the agent framework provides the log data files, which hold raw data of agent behavioral properties accumulated in intervals of 2 s. Hence, in the experiments discussed above, we analyzed the time series of the proposed measure of the whole observation period in intervals of 2 s, as shown in Figure 7 and Figure 8.

The purpose of the on-line mode is to generate alarms quickly in the event of changes in the runtime system using some of time series data of agent behavioral properties. We can calculate the proposed measure in each fixed time period specified in advance of the measurement, and we can obtain a discrete time series of the proposed measure. Moreover, using alarm generation mechanisms, we can detect a time point at which there is a discontinuous change of the measure and issue an alarm at this point. The following simple procedure is an example of alarm generation mechanisms.

[Alarm generation procedure at discrete time point *p*]

- At the discrete time point *p*, the result of observation is given by a time series of observed values; *i.e.*, {$\;v_{0}$, $v_{1}$, ⋯, $v_{p-1}$, $v_{p}$}. Here, $v_{0}=0$.
- The interval of the adjoining observed values, $d_{p}$, is given by $d_{p}\leftarrow v_{p}-v_{p-1}$.
- The maximum value of $d_{t}$ (p≥t≥0) is $d_{max}$.
- The predefined threshold is H (H≥0).
- The score at time point *p*, $S_{p}$, is calculated in this procedure.
- At the initial time point p=0, $S_{0}\leftarrow 0$, $d_{0}\leftarrow 0$, and $d_{max}\leftarrow 0$.
- **if** $d_{p}\geq H\;$
  **then** {**if** $d_{p}>10\times d_{max}$ **then** {$S_{p}\leftarrow 0$; $d_{max}\leftarrow 0$; “Generate Alarm”};
    **if** $d_{p}>d_{max}$ **then** $d_{max}\leftarrow d_{p}$;
    **if** $S_{p-1}>0$ **then** $S_{p}\leftarrow S_{p-1}+1$ **else** $S_{p}\leftarrow 1$ }
  **else** {**if** $|d_{p}|\geq H$
       **then** {$S_{p}\leftarrow 0$; $d_{max}\leftarrow 0$;
           **if** $S_{p-1}>0$ **then** “Generate Alarm”}
       **else** $S_{p}\leftarrow S_{p-1}$ };

The above procedure generates an alarm if a peak of change is detected or $d_{p}$ has a sudden large change exceeding 10 times $d_{max}$. Because $S_{p}$ is reset after the alarm is generated, this procedure can generate alarms many times during a long observation of the runtime system.

**Figure 10:**
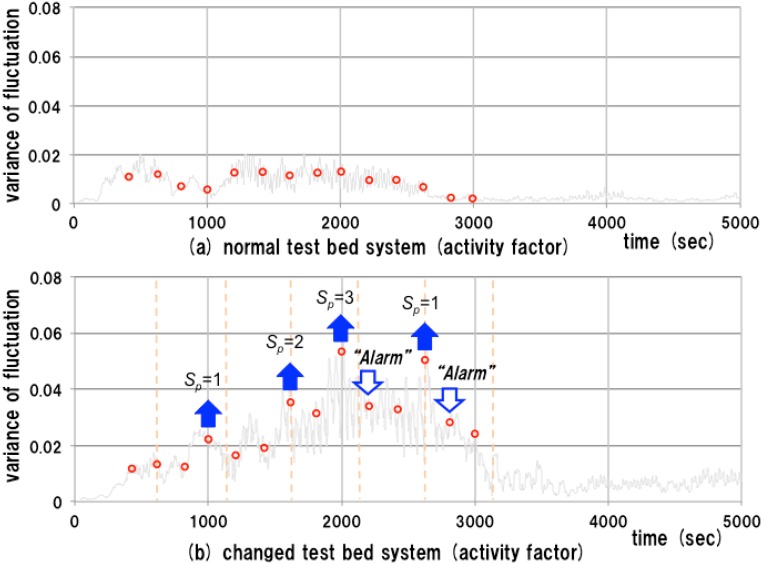
Experiment on an observation in on-line mode.

Figure 10 shows the result of an experiment for the above procedure using some of the data of experiments 1 and 2. The discrete time series of the proposed measure are depicted by circles. In this experiment, we set the fixed observation period to 200 s, and the proposed measure is calculated every 200 s using the short time series of data with length of 40 s. The predefined threshold is set to 0.01.

In the case of the normal test bed system, the observed measure is stable and stays under 0.02, as shown in Figure 10a. No alarm is generated. In the case of the changed system, two alarms are generated in response to discontinuous changes in the observed measure as shown in Figure 10b. The upward arrows show the time points of $d_{p}\geq H$ and the downward arrows show the time points of $|d_{p}|\geq H$. In this case, the alarms are generated in response to large discontinuous changes in the proposed measure.

## 5. Conclusions

A macroscopic measure that responds to abnormal changes in the behavioral property of a multiagent system was proposed in this paper. The proposed measure was designed as the variance of fluctuation in a system’s activity factor according to theoretical analysis of the behavioral model of a multiagent system defined in this paper. To verify basic characteristics of the proposed measure, experiments were conducted using a test bed system based on a repository-based multiagent framework. The results of experiments showed that the proposed measure undergoes an unusual increase in response to abnormal changes in the system. Hence, the proposed measure is considered a measure that can be used to detect undesirable changes in a multiagent system. Further studies on the design of easy-to-use measures for various multiagent applications and the development of a practical measurement environment of runtime systems remain as future work.

## Appendix

### Appendix A. Derivation of the Fokker–Planck Equation

In Equation (17), we introduced two functions:

$$f\left(z,t\right)=(1-z)∙{\text{π}}_{1}\left(z\right)∙\;P(z,\;t)$$

$$g\left(z,t\right)=(1+z)∙{\text{π}}_{2}\left(z\right)∙\;P(z,\;t)$$

Using the Taylor series expansion, these functions are transformed as:

$$f\left(z-\text{ε},t\right)=\sum_{n=0}^{\infty }\frac{1}{n!}∙{\left(-\text{ε}\right)}^{n}∙{\text{π}}_{1}\left(z\right)∙\;\frac{\partial^{n}f(z,t)}{\partial z^{n}}$$

$$g\left(z+\text{ε},t\right)=\sum_{n=0}^{\infty }\frac{1}{n!}∙{\text{ε}}^{n}∙{\text{π}}_{2}\left(z\right)∙\;\frac{\partial^{n}g(z,t)}{\partial z^{n}}$$

The master equation is then transformed as:

(A1) ∂P(z, t)∂t=∑n=0∞1n!∙(−1)n∙εn−1∙ ∂nf(z,t)∂zn+ ∑n=0∞1n!∙εn−1∙ ∂ng(z,t)∂zn

Next, the activity factor *z* is decomposed to the steady-state $z_{0}$ and its fluctuation ξ as:

$$z=\;z_{0}+\sqrt{\text{ε}}∙\text{ξ}$$

From this definition, it follows that:

(A2) $$\text{ξ}=\frac{1}{\sqrt{\text{ε}}}\cdot (z-z_{0})$$

Furthermore, the relations:

$$\frac{\partial }{\partial z}=\frac{\partial \text{ξ}}{\partial z}∙\frac{\partial }{\partial \text{ξ}}+\frac{\partial t^{\prime }}{\partial z}∙\frac{\partial }{\partial t^{\prime }}=\frac{1}{\sqrt{\text{ε}}}\cdot \frac{\partial }{\partial \text{ξ}}$$

and:

(A3) $$\frac{\partial }{\partial t}=\frac{\partial \xi }{\partial t}∙\frac{\partial }{\partial \text{ξ}}+\frac{\partial t^{\prime }}{\partial t}∙\frac{\partial }{\partial t^{\prime }}=\frac{1}{\sqrt{\text{ε}}}\cdot \frac{\partial z_{0}}{\partial t}∙\frac{\partial }{\partial \text{ξ}}+\frac{\partial }{\partial t^{\prime }}$$

are introduced. Using Equations (A2) and (A3), the master Equation (A1) is transformed as:

(A4) $$\frac{\partial }{\partial t}-\frac{1}{\sqrt{\text{ε}}}\cdot \frac{\partial z_{0}}{\partial t}∙\frac{\partial }{\partial \text{ξ}}\;P(\text{ξ},t)\;=\;\sum_{n=0}^{\infty }\frac{1}{n!}∙{\left(-1\right)}^{n}∙{\text{ε}}^{\frac{n}{2}-1}∙\;\frac{\partial^{n}f\left(z_{0}\left(t\right)+\sqrt{\text{ε}}∙\text{ξ},\;t\right)}{\partial {\text{ξ}}^{n}}\;+\;\sum_{n=0}^{\infty }\frac{1}{n!}∙{\text{ε}}^{\frac{n}{2}-1}∙\;\frac{\partial^{n}g\left(z_{0}\left(t\right)+\sqrt{\text{ε}}∙\text{ξ},\;t\right))}{\partial {\text{ξ}}^{n}}\;=\;\sum_{n=0}^{\infty }\frac{1}{n!}∙{\left(-1\right)}^{n}∙{\text{ε}}^{\frac{n}{2}-1}∙\;\frac{\partial^{n}}{\partial {\text{ξ}}^{n}}[\;\sum_{m=0}^{\infty }\frac{1}{m!}∙{\left({\text{ε}}^{\frac{1}{2}}\cdot \text{ξ}\right)}^{m}∙\;\frac{\partial^{m}f\left(z_{0}\left(t\right),\;t\right)}{\partial {z_{0}}^{m}}\;]\;+\;\sum_{n=0}^{\infty }\frac{1}{n!}∙{\text{ε}}^{\frac{n}{2}-1}∙\;\frac{\partial^{n}}{\partial {\text{ξ}}^{n}}[\;\sum_{m=0}^{\infty }\frac{1}{m!}∙{\left({\text{ε}}^{\frac{1}{2}}\cdot \text{ξ}\right)}^{m}∙\;\frac{\partial^{m}g\left(z_{0}\left(t\right),\;t\right)}{\partial {z_{0}}^{m}}\;]$$

Using Equation (A4), the evolution of $z_{0}$ is given by the smallest terms of ε; *i.e.*, $\frac{1}{\sqrt{\text{ε}}}$.

Left hand side of Equation (A4): $-\frac{\partial z_{0}}{\partial t}∙\frac{\partial P(\text{ξ},\;t)}{\partial \text{ξ}}$.

Right hand side of Equation (A4): in the case of n=1 and m=0:

$$-\frac{\partial }{\partial \text{ξ}}∙\left(1-z_{0}\right)∙{\text{π}}_{1}\left(z_{0}\right)∙P\left(\text{ξ},t\right)+\;\frac{\partial }{\partial \text{ξ}}∙(1+z_{0})∙{\text{π}}_{2}\left(z_{0}\right)∙P\left(\text{ξ},t\right)\;=\;-[(1-z_{0})∙{\text{π}}_{1}\left(z_{0}\right)-\;(1+z_{0})∙{\text{π}}_{2}\left(z_{0}\right)]∙\frac{\partial P(\text{ξ},t)}{\partial \text{ξ}}$$

Hence, the evolution of $z_{0}$ is:

(A5) ∂z0∂t= (1−z0)∙π1(z0)− (1+z0)∙π2(z0)

On the other hand, the Fokker–Planck equation is derived from the terms of ${\text{ε}}^{0}$ in Equation (A4).

Left hand side of Equation (A4): $\frac{\partial P(\text{ξ},\;t)}{\partial t}$.

Right hand side of Equation (A4): in the case of n=2 and m=0, and the case of n=1 and  m=1:

$$\frac{1}{2}∙\;\frac{\partial^{2}f\left(z_{0},\;t\right)}{\partial {\text{ξ}}^{2}}-\;\frac{1}{\sqrt{\text{ε}}}∙\;\frac{\partial }{\partial \text{ξ}}∙\left[\sqrt{\text{ε}}∙\text{ξ}∙\frac{\partial f\left(z_{0},t\right)}{\partial z_{0}}\right]\;+\;\frac{1}{2}∙\;\frac{\partial^{2}g\left(z_{0},\;t\right)}{\partial {\text{ξ}}^{2}}+\;\frac{1}{\sqrt{\text{ε}}}∙\;\frac{\partial }{\partial \text{ξ}}∙\left[\sqrt{\text{ε}}∙\text{ξ}∙\frac{\partial g\left(z_{0},t\right)}{\partial z_{0}}\right]\;=\frac{1}{2}∙[(1-z_{0})∙{\text{π}}_{1}\left(z_{0}\right)+(1+z_{0})∙{\text{π}}_{2}\left(z_{0}\right)]∙\frac{\partial^{2}P(\text{ξ},t)}{\partial {\text{ξ}}^{2}}\;-\;\frac{\partial }{\partial z_{0}}∙[(1-z_{0})∙{\text{π}}_{1}\left(z_{0}\right)-(1+z_{0})∙{\text{π}}_{2}\left(z_{0}\right)]∙\frac{\partial [\text{ξ}∙P\left(\text{ξ},t\right)]}{\partial \text{ξ}}$$

Hence, the Fokker–Planck equation is:

(A6) ∂P(ξ,t)∂t= − ∂∂z0∙[(1−z0)∙π1(z0)−(1+z0)∙π2(z0)]∙∂[ξ∙P(ξ,t)]∂ξ+ 12∙[(1−z0)∙π1(z0)+(1+z0)∙π2(z0)]∙∂2P(ξ,t)∂ξ2

### Appendix B. Derivation of the Variance of Fluctuation from the Fokker–Planck Equation

A general form of the Fokker–Planck equation with respect to fluctuation $\hat{u}$ is given as:

$$\hat{u}=\text{\{}u_{i}\;\text{| i}=1,2,\cdots ,n\}$$

(B1) $$\frac{\partial P(\hat{u},t)}{\partial t}\;=\;-\;\frac{\partial }{\partial \hat{u}}∙\left[K∙\hat{u}∙P\left(\hat{u},t\right)\right]+\frac{1}{2}∙\frac{\partial^{2}}{\partial {\hat{u}}^{2}}∙[D∙P\left(\hat{u},t\right)]$$

The variance of fluctuation can be derived using Equation (B1). Considering the definitions:

(B2) $${\text{σ}}_{ij}^{2}\equiv \int^{\text{​}}u_{i}∙u_{j}∙P\left(\hat{u},t\right)du$$

(B3) $${\text{σ}}^{2}=<{\left(\hat{u}-\;<\hat{u}>\right)}^{2}>\;=<\hat{u}∙\hat{u}>-<\hat{u}{>}^{2}$$

We have the evolution of variance as:

(B4) $$\frac{\partial {\text{σ}}^{2}}{\partial t}\;=\int^{\text{​}}\hat{u}∙\hat{u}∙\frac{\partial }{\partial t}P\left(\hat{u},t\right)du-2∙<\hat{u}>\int^{\text{​}}\hat{u}∙\frac{\partial }{\partial t}P\left(\hat{u},t\right)du$$

The first term of Equation (B4) can be rewritten using the Fokker–Planck equation:

(B5) $$\int^{\text{​}}\hat{u}∙\hat{u}∙[-\frac{\partial }{\partial \hat{u}}\left\{K∙\hat{u}∙P\left(\hat{u},t\right)\right\}+\frac{1}{2}∙\frac{\partial^{2}}{\partial {\hat{u}}^{2}}\{D∙P\left(\hat{u},t\right)\}]d\hat{u}\;=-K\int^{\text{​}}\hat{u}∙\hat{u}∙\frac{\partial }{\partial u}\left\{\hat{u}∙P\left(\hat{u},t\right)\right\}d\hat{u}+\frac{1}{2}∙D∙\int^{\text{​}}\hat{u}∙\hat{u}∙\frac{\partial^{2}}{\partial {\hat{u}}^{2}}P\left(\hat{u},t\right)d\hat{u}\;=\;-K∙[\hat{u}∙\hat{u}∙\hat{u}∙P\left(\hat{u},t\right)-2∙\int^{\text{​}}\hat{u}∙\hat{u}∙P\left(\hat{u},t\right)d\hat{u}\;+\;\frac{1}{2}∙D∙[\;\hat{u}∙\hat{u}∙\frac{\partial }{\partial \hat{u}}P\left(\hat{u},t\right)-2∙\hat{u}∙P\left(\hat{u},t\right)+2]\;=2∙K∙<\hat{u}∙\hat{u}>+D$$

Here, we suppose that $P\left(\hat{u},t\right)\to 0\;\text{and}\;\frac{\partial }{\partial u}P\left(\hat{u},t\right)\to 0$ when t→∞.

The second term is rewritten as:

(B6) $$\int^{\text{​}}\hat{u}∙[-\frac{\partial }{\partial \hat{u}}\left\{K∙\hat{u}∙P\left(\hat{u},t\right)\right\}+\frac{1}{2}∙\frac{\partial^{2}}{\partial {\hat{u}}^{2}}\{D∙P\left(\hat{u},t\right)\}]d\hat{u}\;=-K\int^{\text{​}}\hat{u}∙\frac{\partial }{\partial \hat{u}}\left\{\hat{u}∙P\left(\hat{u},t\right)\right\}d\hat{u}+\frac{1}{2}∙D∙\int^{\text{​}}\hat{u}∙\frac{\partial^{2}}{\partial {\hat{u}}^{2}}P\left(\hat{u},t\right)d\hat{u}\;=\;-K∙[\hat{u}∙\hat{u}∙P\left(\hat{u},t\right)-\int^{\text{​}}\hat{u}∙P\left(\hat{u},t\right)d\hat{u}\;+\;\frac{1}{2}∙D∙[\;\hat{u}∙\frac{\partial }{\partial \hat{u}}P\left(\hat{u},t\right)-\int^{\text{​}}\frac{\partial }{\partial \hat{u}}P\left(\hat{u},t\right)d\hat{u}]\;=K∙<\hat{u}>$$

From Equations (B5) and (B6), the evolution of variance is given as:

(B7) $$\frac{\partial {\text{σ}}^{2}}{\partial t}\;=2∙K∙<\hat{u}∙\hat{u}>+D-2∙<\hat{u}>∙K<\hat{u}>\;=2∙K∙{\text{σ}}^{2}+D$$

In Fokker–Planck Equation (18), the parameters in Equation (B7) are given as:

(B8) $$K=\frac{\partial }{\partial z_{0}}∙[(1-z_{0})∙{\text{π}}_{1}\left(z_{0}\right)-(1+z_{0})∙{\text{π}}_{2}\left(z_{0}\right)]$$

(B9) $$D=(1-z_{0})∙{\text{π}}_{1}\left(z_{0}\right)+(1+z_{0})∙{\text{π}}_{2}\left(z_{0}\right)$$

From Equations (B7)–(B9), we have Equation (21).
